# Modulation of the Complement System by Neoplastic Disease of the Central Nervous System

**DOI:** 10.3389/fimmu.2021.689435

**Published:** 2021-10-04

**Authors:** Steven K. Yarmoska, Ali M. Alawieh, Stephen Tomlinson, Kimberly B. Hoang

**Affiliations:** ^1^ Department of Neurosurgery, Emory University School of Medicine, Atlanta, GA, United States; ^2^ School of Electrical and Computer Engineering, Georgia Institute of Technology, Atlanta, GA, United States; ^3^ Department of Microbiology and Immunology, Medical University of South Carolina, Charleston, SC, United States

**Keywords:** complement, brain, cancer, glioblastoma, glioma, metastasis, leptomeningeal, radiation

## Abstract

The complement system is a highly conserved component of innate immunity that is involved in recognizing and responding to pathogens. The system serves as a bridge between innate and adaptive immunity, and modulation of the complement system can affect the entire host immune response to a foreign insult. Neoplastic diseases have been shown to engage the complement system in order to evade the immune system, gain a selective growth advantage, and co-opt the surrounding environment for tumor proliferation. Historically, the central nervous system has been considered to be an immune-privileged environment, but it is now clear that there are active roles for both innate and adaptive immunity within the central nervous system. Much of the research on the role of immunological modulation of neoplastic disease within the central nervous system has focused on adaptive immunity, even though innate immunity still plays a critical role in the natural history of central nervous system neoplasms. Here, we review the modulation of the complement system by a variety of neoplastic diseases of the central nervous system. We also discuss gaps in the current body of knowledge and comment on future directions for investigation.

## Introduction

The central nervous system (CNS) has been traditionally described as an immune-privileged environment. However, cumulative data over the past two decades have demonstrated that within the brain parenchyma as well as at the CNS endothelial surfaces, robust adaptive and innate immune responses can be elicited during both normal development and disease processes. In the context of neoplastic disease, inflammatory and immune mechanisms have been implicated in disease progression, response to systemic and local therapy, neurodegeneration and cerebral edema. The complement system, a component of the innate immunity, has been a major recent focus in the area of neuroscience given the role of complement proteins in early detection of stress signals, orchestrating both innate and adaptive responses and driving long-term neuroplasticity (1). In this work, we review the recent updates on the role of different complement components in the pathogenesis of neoplastic diseases of the CNS, both primary and metastatic, and the implications of this role in therapeutic interventions.

## The Complement System

**Figure 1:**
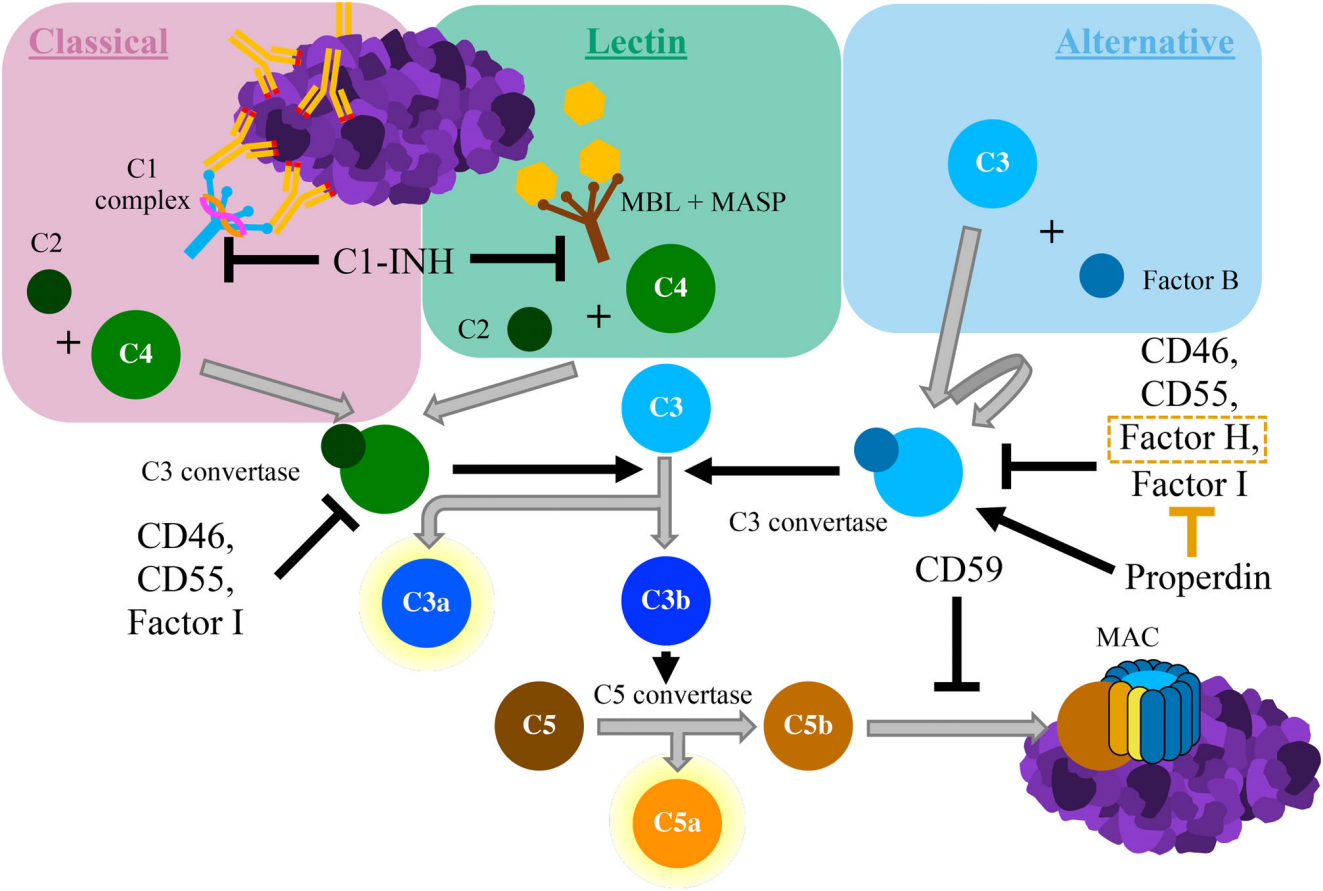
Schematic diagram of the complement cascade. The classical pathway begins with the binding of the C1 complex to antigen-antibody complexes containing IgG or IgM. The lectin pathway is triggered by the binding of mannose-binding lectin (MBL), which is bound to proteolytic MBL-associated serine proteases (MASPs), to carbohydrate moieties. Both of these pathways result in cleavage of C2 and C4 to form C3 convertase. The alternative pathway starts with the autoactivation of C3, typically *via* direct binding of C3 to pathogens. This creates a unique version of C3 convertase that involves the cleavage of Factor B All complement pathways converge with the cleavage of C3 into C3a and C3b. C3b associates with C3 convertase, which cleaves C5 into C5a and C5b. The anaphylatoxins C3a and C5a have potent proinflammatory effects. C5b deposits on cell surfaces, where it forms the membrane attack complex (MAC), which induces cell lysis. Various inhibitory proteins disrupt the complement cascade, as labelled within the figure above.

The complement system is an integral component of innate immunity. It was discovered in the late nineteenth century when it was shown to “complement” the ability of heat-stabile antibodies to kill bacteria (2). Similar to this seminal *in vitro* experiment, the *in vivo* complement system both directly responds to pathogens and indirectly recruits the adaptive immune system to assist in host defense (3). The complement system can be activated through one of three distinct pathways: the classical pathway, the lectin pathway, and the alternative pathway (4) (see **Figure 1**). The lectin pathway is triggered by recognition of conserved molecular patterns expressed on cells surfaces, whereas the classical pathway is triggered by antigen-bound IgG or IgM antibodies (5). The alternative pathway can be spontaneously activated on surfaces of foreign cells that lack complement regulators, and it also serves as an amplification loop for complement activation by other pathways (6). Through the sequential cleavage of unique zymogen proteins, all pathways converge at the cleavage and activation of complement protein C3 *via* the C3 convertase enzyme. Activation of protein C3 leads to the production of C3 opsonins, the anaphylatoxins C3a and C5a, and ultimately the membrane attack complex (MAC; C5b-9). Complement opsonins (C1q, C3b/d) deposit on surfaces of cells to tag them for phagocytosis and serve as activators of immune cells and microglia (7, 8). The complement anaphylatoxins have a multitude of proinflammatory effects including leukocyte recruitment, vasodilation, and the induction of mast cell degranulation and neutrophil oxidative burst. The MAC assembles in cell membranes, disrupting osmotic regulation and leading to cell lysis.

Complement opsonins also directly engage the development of B cell immunity *via* CD21 and CD35. On B cells, activation of these receptors lowers the threshold for B-cell receptor activation (9). On follicular dendritic cells, their expression leads to the retention of C3-coated particles for downstream presentation to B cells (10). Additionally, the complement system impacts T cell immunity. As in B cell immunity, C3 is thought to play a role in antigen presentation by marking foreign materials for APCs to phagocytose and present to T cells. However, studies on the role of C3 in the development of T cell immunity are equivocal (11–15), suggesting that this effect is antigen dependent. Murine studies have implicated C3a and C5a anaphylatoxins as chemoattractants that promote T cell recruitment either *via* direct effects on T cells or *via* indirect effects on the cytokine response of APCs (16, 17). Complement also provides negative feedback on the immune response through the induction of a regulatory phenotype in T cells through the co-stimulation of CD3 and CD46 by complement proteins C3b and C4b (3, 18).

A variety of mechanisms serve to regulate the complement system. The anaphylatoxins C3a and C5a are rapidly reduced *via* the removal of a C-terminal arginine, which dramatically reduces their biological activity (19). Similarly, C3b and C4b are cleaved by the serine protease Factor I into inactive metabolites with regards to complement activation, although the breakdown products still serve as opsonins. Several of the C3b breakdown products are bound by complement receptors (CR2, CR3, and CR4) that function within both the adaptive and innate immune systems (20). Factor H also accelerates the breakdown of C3 convertase in the alternative pathway (21). C3 convertase can be inhibited by the effects of decay acceleration factor (DAF; CD55) and C4 binding protein, as well. Upstream of the convergent complement components, C1 inhibitor (C1-INH) inactivates C1r and C1s as well as MASP2 (mannan-binding serine protease 2) (22), which are involved in the classical and lectin pathways, respectively. Downstream of the convergence of the complement pathways, formation of the MAC is negatively regulated by S protein, vimentin, and CD59 (4).

Properdin is the only known positive regulatory protein of the complement cascade (23). It binds to and stabilizes C3 convertase in the alternative pathway (24). Additionally, it inhibits the cleavage of C3b by Factor H (25, 26). A family of proteins structurally similar to Factor H, known as Factor H related proteins, also exist (27). However, the roles of these proteins within the canonical complement cascade are still under active investigation.

## Complement in the Central Nervous System

Circulating complement proteins can extravasate into the CNS when a pathologic inflammatory, infectious, or vascular process disrupts the integrity of the BBB (28). However, complement proteins can be found within the CSF of patients with an intact BBB, and recent evidence has shown that CNS-resident cells, including neurons and glial cells, are capable of producing nearly all complement proteins independent of serum derived factors (29). *In vitro* experiments demonstrated that astrocytes express complement proteins C2 through C9 and Factor B as well as regulatory proteins, such as Factor H and Factor I (30–32). Astrocytes have also been shown to express C5a receptor 1 (C5aR1), as well as the membrane regulatory proteins CD46, CD55, CD59, and CR1 (33). Similarly, both oligodendrocytes (34) and neurons (35, 36) have been shown to express complement proteins of the classical and alternative pathways. A subset of these genes are also expressed by microglia (37, 38).

In the healthy CNS, microglia recognize complement proteins C1q and C3 opsonins on synapses to be eliminated in the process of synaptic remodeling (39, 40). Low concentrations of MAC have also been shown to be protective for oligodendrocytes against apoptosis secondary to caspase-3 activation (41, 42). In pathologic states, the activation of complement proteins has been linked to the exacerbation of neuroinflammatory and neurodegenerative pathways in multiple sclerosis (43), stroke (1), and neurodegenerative diseases (44–46).

Neoplastic diseases within the CNS can modulate the expression and function of complement proteins in order to facilitate a pro-growth niche. The role of complement in cancer is complex, and evidence exists for both activation and inhibition of the complement cascade to result in tumorigenesis. Historically, due to the role of complement in immune surveillance (47, 48), it was thought that complement activation protected against neoplastic disease. However, more recent literature has demonstrated that complement activation within primary tumor microenvironments can enhance neoplastic growth (48, 49). The CNS provides a unique microenvironment of its own, and a diverse number of primary and secondary neoplasms can invade the CNS. Although the direct effects of complement on metastatic disease may be conserved regardless of their location, studies of complement modulation in tumors located outside the CNS may not directly translate to those located within the CNS when it comes to complement-dependent changes in the tumor microenvironment.

In this review, we discuss the role of different complement components in the pathology of CNS tumors and their implication for therapeutic interventions. A summary of the studies of complement modulation in CNS tumors, organized by disease state, can be found in **Table 1**. Particular focus will be placed on malignant neoplastic diseases that continue to have poor prognosis (e.g., glioma, metastasis, and leptomeningeal disease) where complement modulation may play a significant therapeutic role.

**Table 1:** Complement expression in brain neoplasms.

Component	Change	Material	Source	Disease	Model	Reference
C1q, C1-INH	Overexpressed	RNA, Protein	Tumor cells	Glioma	*In vitro* human patient-derived cells, *In vivo* rat glioma	(50)
C1q	Overexpressed	RNA, Protein	Tumor cells	Glioma	Human glioma samples	(51)
C1r, C1s	Overexpressed	RNA	Tumor cells	Glioma	Human glioma samples	(52)
MASP-1/3	Overexpressed	RNA, Protein	Tumor cells	Glioma	*In vitro* immortalized human and rodent cells	(53, 54)
C3	Overexpressed	RNA	Tumor cells	Glioma	*In vitro* human patient-derived cells, In vivo mouse xenograft	(55)
Factor H	Overexpressed	Protein; RNA, Protein	Tumor cells	Glioma	*In vitro* immortalized human cells	(56, 57)
FHL-1	Overexpressed	RNA, Protein	Tumor cells	Glioma	*In vitro* immortalized human cells	(57)
FHR5	Overexpressed	Protein	Tumor cells	Glioma	*In vitro* human patient-derived cells	(58)
CD55	Overexpressed	Protein	Tumor cells	Glioma	*In vitro* immortalized human cells	(59)
CD59	Overexpressed	Protein	Tumor cells	Glioma	Human glioma samples, Glioma cell lines	(60)
CD59	Overexpressed	Protein	Tumor cells	Glioma	*In vitro* immortalized human cells	(59)
C1q, Factor H, C3aR, C5aR	Overexpressed	RNA	Tumor cells	Glioma	Human glioma samples	(61)
C1q	Polymorphism	DNA	Tumor cells	Metastasis	Human tissue samples (breast)	(62)
CD59	Overexpressed	Protein	Tumor cells	Metastasis	*In vitro* immortalized human cells (prostate)	(63)
C1q, C3, C5, CD46	Mutated	DNA	Tumor cells	Metastasis	Matched primary and metastatic human tissue (breast, lung, kidney)	(64)
C3	Overexpressed	RNA, Protein	Tumor cells, CSF	LMD	*In vivo* LMD-derived *in vitro* culture (breast, lung)	(65)
C2, C3, MAC	Overexpressed	RNA, Protein	CSF	LMD	Human CSF samples (melanoma)	(66)
C1q, Factor H	Overexpressed	Protein	CSF	LMD	Human CSF samples (lymphoma)	(67)
C1q, C2, CR1, CFB, C3aR	Upregulated	RNA	Brain tissue	Radiation Induced Brain Injury	Fractionated whole-brain irradiation in NHP	(68)
CR3	Activated	Protein	Brain tissue	Radiation Induced Brain Injury	*In vivo* mouse	(69)

## Complement in Neoplastic Diseases of the CNS

### Complement in Glioma and Glioblastoma Multiforme

A “glioma” refers to any tumor that is thought to be of glial cell origin (70), including astrocytomas, oligodendrogliomas, ependymomas, and other rare or mixed subtypes within this family (71). High-dose radiation exposure and genetic syndromes (e.g., Li-Fraumeni syndrome, neurofibromatosis, tuberous sclerosis) are the only proven risk factors for the development of gliomas (70). Glioblastoma (i.e., WHO Grade IV astrocytoma) is the most common glioma and carries the worst prognosis. The specific inflammatory microenvironment of glioblastoma contributes to its aggressiveness, recurrence, and resistance to treatment (72). Although much of the research on immune modulation in glioma progression has focused on the adaptive immune system, innate immunity and complement also play roles in shaping the tumor microenvironment and orchestrating both adaptive and innate immune responses.

#### Classical Pathway in Glioma

Consistent with the paradigm of immune evasion, current research on complement modulation in glioblastoma has focused on the classical pathway and the C1 complex. In both *in-vitro* and *in-vivo* models, glioblastoma was shown to upregulate the expression of the C1-INH protein that prevents the assembly of the C1 complex and inhibits the initiation of complement activation *via* the classical pathway (50). In synchrony with these findings, human glioblastoma tumor samples have been shown to demonstrate upregulation of the C1 complex proteins (C1q, C1s) as well as C1-INH protein (50). However, in complement pathology, mRNA upregulation does not necessarily correlate with function, given the need for proteolytic cleavage for complement activation. An increase in C1-INH effect results in upregulation of proximal classical pathway genes, which may explain the co-increase in expression of both C1q/C1s and C1-INH genes. Following these observations, the use of C1-INH as a treatment in a murine non-orthotopic model of glioblastoma demonstrated reduced tumor growth and prolonged host survival (73) (see **Table 2** regarding preliminary targeted therapeutics). Serum analysis of rats in these studies showed a decrease in circulating IL-1β, one of the major proinflammatory cytokine downstream of the inflammasome complex that is produced by glioblastoma cells (77).

**Table 2:** Exogenous complement modulation as treatment in brain neoplasms.

Tumor	Model	Intervention	Outcome	Reference
Glioblastoma multiforme (GBM)	*In vivo* rat GBM	Anti-C1-INH monoclonal antibody	Increased survival, decreased tumor growth	(73)
Metastasis	*In vitro* prostate metastatic cells	Anti-CD59 antibody	Complement-mediated cytolysis	(74)
Leptomeningeal disease (LMD)	*In vivo* mouse MDA 231 (breast) LLM (lung) PC9 (lung)	C3 shRNA knockdown	Reduced LMD growth	(65)
Leptomeningeal disease (LMD)	Human peripheral blood (lymphoma)	Anti-CD20 monoclonal antibody (rituximab)	C3b upregulation, NK cell inactivation	(75)
Leptomeningeal disease (LMD)	Human CSF (lymphoma)	Anti-CD20 monoclonal antibody (rituximab)	C3 and MAC upregulation	(76)

In addition to its role as an initiator of complement activation, the C1 complex also includes the C1q protein. The C1q protein has an independent function of opsonization and clearance of apoptotic blebs, a process that is implicated in immune tolerance (78) and that has been described in the settings of autoimmunity (79, 80), angiogenesis (81), and tumorigenesis (80, 82). Although C1-INH binds to C1r and C1s, it does not bind to or inactivate C1q. Like C1r and C1s (52), C1q is also upregulated in response to the increased levels of C1-INH in glioblastoma (50). There is a greater than 4-fold change in C1q expression observed in human glioblastoma tissue samples (50), and genomic expression of C1q is positively correlated with unfavorable prognosis for patients in national glioma databases (51, 61). The tumorigenic effects of C1q are thought to be a product of both increased angiogenesis, promoting immune tolerance by clearance of antigenic products of tumor invasion, as well as direct promotion of adhesion and proliferation through integration within the extracellular matrix (82). C1q expression has been shown to activate canonical WNT signaling pathways (83), which have been shown to facilitate epithelial-to-mesenchymal transition of glioblastoma cells *in vitro* (84). Conversely, C1q has also been shown to inhibit tumor growth in models of breast (85), ovarian (86), and prostate cancers (87). It is thought that this inhibitory phenotype is secondary to increased tumor cell apoptosis. In the CNS, C1q is recognized by microglia during the process of synaptic pruning (39, 40), a mechanism that can also be carried out by astrocytes (88, 89). Additionally, C1q has been shown to induce a tumorigenic phenotype in tumor associated macrophages (90), a process that may extend to microglia (91, 92). Therefore, these findings suggest that the pro-growth and pro-survival effects of C1q on astroglial cells is preserved during malignant transformation as seen in primary brain tumors (i.e., gliomas). However, this may also explain why C1q has mostly a pro-apoptotic and tumor inhibitory effect on brain metastases as opposed to its effect on primary brain tumor cells.

#### Lectin Pathway in Glioma

The lectin pathway is initiated by the binding of mannose-binding lectin (MBL), which is structurally and functionally similar to C1q of the classical pathway (93). MBL predominantly binds to carbohydrate patterns, such as those found on pathogens (94–96) or IgM (97). When this binding occurs, the process activates MBL-associated serine proteases (MASPs) that subsequently cleave downstream complement proteins to propagate activation of the cascade (98). Three distinct MASPs have been described (98, 99), each of which has unique protease activity. *In vitro* studies of human and rat glioma cells have demonstrated their ability to secrete high levels of MASP-1 and MASP-3 (53, 54). It remains unclear whether there is a unique role for these proteases in disease progression or whether their upregulation is in response to upregulated C1-INH, which can also bind and inhibit the function of MASPs (72). Additional research is necessary to elucidate the role of MASP-1/3 in the natural history of glioma.

#### Alternative Pathway and Complement Regulators in Glioma

The alternative complement pathway plays a pivotal role in the amplification of complement activation and downstream effectors of complement by its feedforward effect on complement protein C3 activation. The alternative pathway is capable of promoting pathological levels of complement activation that may be able to evade the endogenous complement inhibitory proteins on surfaces of neurons and glia, as has been shown in ischemic and traumatic brain injury (100–103). Glioblastoma cells have demonstrated the ability to synthesize complement proteins, including C3 (55) and its receptor (61). Additionally, Factor H, which is expressed by astrocytes in the CNS, is a major fluid phase inhibitor of the alternative complement pathway. *In vitro* studies have demonstrated that glioblastoma cells can produce Factor H (56, 57), a result that has been supported by studies on *ex vivo* patient samples (61). Similarly, FH-like protein 1 (FHL-1) (57), a truncated form of Factor H that retains inhibitory activity against C3, is secreted by glioblastoma cells *in vitro* (21, 104). In addition to Factor H, glioblastoma cells were also shown to have upregulated expression of complement factor H related protein 5 (FHR5) (58). However, the physiological and pathological functions of the newly described FHR5 are still controversial (58, 105, 106).

Membrane-bound CD55 both prevents the assembly of and causes the dissociation of both C3 and C5 convertase (4, 107). Downstream of C3 and C5 activation, the cell-surface protein CD59 inhibits the formation of the terminal MAC by preventing C9 protein polymerization (108). Human-derived glioblastoma cell lines grown *in vitro* have been shown to have upregulated CD55 and CD59 (59, 60), which may be correlated with mutations in the p53 tumor suppressor gene (109). Expression of CD55 and CD59 could theoretically inhibit complement activation at the surface of growing GBM tumors *in vivo*, and CD59 overexpression has been observed in human glioma samples (60). However, lack of CD55 expression was found to be correlated with poor prognosis in solid tumors, such as breast cancer (110), and so further research is necessary to elucidate the effects of these proteins in glioblastoma at the organismal level. Moreover, the CD55 expression observed in GBM may be a multifactorial phenomenon.

Collectively, the current literature on the role of complement in glioblastoma support an overall tendency of the tumor to suppress the activation of the C3 protein and downstream effectors, while simultaneously preserving the functions of the C1q opsonins necessary for enhanced immune tolerance during tumor progression. This is in contrast to other primary cancers, such as cervical (111), breast (112), and lung (113), in which the complement system is activated in preclinical models to promote immune tolerance through the enhanced production of regulatory T cells (Tregs) and suppression of conventional T cells (114). Tregs are a major source of immune tolerance in glioblastoma, as Treg infiltration directly correlates to the WHO tumor grade (115), but the interplay between the complement system and Tregs within the context of glioma has yet to be conclusively elucidated.

### Complement in Brain Metastasis

Brain metastases are the most common type of intracranial tumor in adults (116). The most common sources of brain metastases are from primary breast, lung, and skin (i.e., melanoma) cancers (117). The “seed and soil” hypothesis defines metastasis to the brain, whereby circulating tumor cells attach to endothelial cells in brain vasculature, extravasate, and proliferate within the brain parenchyma (118). The majority of cancer cells that successfully extravasate into the brain parenchyma are ill-adapted to this microenvironment and subsequently die (119). However, this microenvironment imposes a selective pressure on migrating cancer cells that ultimately selects successful clones to seed the brain (120–122). A major component of this selective process is an ability of the metastasizing cell to evade the immune response (123). As a link between innate and adaptive immunity, complement modulation is one potential avenue for metastatic tumor cells to initiate this process.

#### Hypercoagulability in Metastatic Disease

Many metastatic cancers also present with thrombosis secondary to hypercoagulation (124). Modulation of the complement cascade is one potential factor contributing to this observed hypercoagulability in cancer (125–127). Pathway analysis has demonstrated a concomitant enrichment of proteins in the complement and coagulation pathways for brain metastases in breast, kidney, and lung cancer (64). Complement and coagulation are intrinsically related. For example, several complement proteins interact with tissue factor (TF; Factor III) and TF-bearing microparticles produced by tumor cells (128, 129). Hypercoagulation and thrombosis impact the metastatic process *via* dysregulation of the immune system. Peritumoral thrombosis has been shown to recruit inflammatory monocytes that promote metastatic tumor cell survival (130). Additionally, thrombi may induce a tumorigenic phenotype in neutrophils that helps to maintain a metastatic niche (131). Thrombin has also been shown to inhibit the expression of tumor suppressor genes within tumor cells (132). Furthermore, platelets can release growth factors that stimulate tumor growth, angiogenesis, and epithelial-to-mesenchymal transition (133, 134). Platelets have also demonstrated the ability to inhibit NK cell lysis of tumor cells, as well (135, 136). Moreover, it has been hypothesized that platelets, fibrin, and thrombin can shield circulating tumor cells from shear forces as they extravasate (127, 137). Clinically, anticoagulation has been shown to decrease the incidence of lung metastases (138, 139), but the effect of systemic anticoagulation on the development of brain metastasis remains unclear.

#### Breast Cancer Metastasis

In this context, a single nucleotide polymorphism (SNP) within the C1q protein has been associated with an increased rate of breast cancer metastasis (62, 64). This association is even more pronounced for metastatic sites associate with hematogenous spread, such as the brain. The C1q protein is composed of six trimers of C1qA, C1qB, and C1qC chains (140). Among all of the known polymorphisms in these components, the only SNP within a coding region is at the 276^th^ position of C1qA. Patients with either a homogenous or heterogeneous A-to-G polymorphism in the C1qA chain were found to have an increased risk of developing metastasis to bone, brain, or liver compared to patients without this polymorphism, even after adjusting for node and receptor status. Patients with the SNP also had a reduced time to develop these extranodal metastases. Previous studies have associated the “A” polymorphism with the development of systemic lupus erythematosus (141). This may imply that enhanced solid organ metastases with the “G” polymorphism is secondary to decreased immune activity, potentially due to reduced ability for the C1 complex to bind to and clear circulating tumor cells.

#### Prostate Metastasis

Similar to the case of glioma, malignant prostate cells have been shown to overexpress CD59 *in vitro* (63). Prostasomes are secretory vesicles produced by prostate cells, and they have been shown to contain the complement regulatory proteins CD46 and CD59 (142). In the case of prostate cancer, these prostasomes are secreted into the tumor microenvironment as opposed to the seminal plasma, which may contribute to the immune evasion and malignant potential of the growing tumor. A study by Babiker et al. demonstrated that prostasomes produced from prostate cancer cells contained a higher concentration of CD59 than those produced from benign prostate cells (63). Additionally, this study demonstrated that tumor-derived prostasomes were able to transfer CD59 to the surface of malignant cells *in vitro*. Prostasomes from brain-metastasis-derived DU145 cells exhibited the highest average CD59 content and one of the largest inhibitory effects on complement. These cells have been shown to be sensitive to complement-mediated cytolysis in the presence of a CD59-neutralizing antibody (74) (see **Table 2**), reinforcing the role of this molecular interaction. Follow-up studies are necessary to further elucidate the role of CD59 expression on the metastatic potential of prostate cells *in vivo*.

### Primary CNS Lymphoma

In CNS lymphomas, there is a growing body of evidence to support a role for complement in the pathogenesis of disease. Analysis of the CSF of patients with primary CNS lymphoma (PCNSL) demonstrates an upregulation of proteins associated with the innate immune system, including complement proteins C1q and Factor H (67). The upregulation of Factor H may contribute to overall suppression of the complement system and promotion of a tumorigenic microenvironment similar to studies in glioma, as discussed previously. However, the upregulation of C1q has been associated with a variety of downstream effects in different cancers and requires further exploration in PCNSL. Complement suppression being tumorigenic in PCNSL would be consistent with murine studies in non-CNS lymphoma that demonstrated attenuated tumor progression with complement activation (143).

Additionally, the intrathecal administration of rituximab, a monoclonal antibody against CD20 on B cells, has been shown to induce complement-dependent tumor cytotoxicity in PCNSL (76, 144) (see **Table 2**). In one clinical trial, complement proteins C3 and C5b-9 were found to be reproducibly upregulated within the CSF in response to the intrathecal administration of rituximab (76). Paradoxically, the constitutive activation of C3 within the CSF was correlated with a worse overall prognosis. The authors of this study hypothesize that this phenomenon may be due to NK cell inactivation by C3b, which has been previously demonstrated in the context of rituximab therapy (75). Alternatively, this may be related to immune dysregulation secondary to the role of C3a as a chemoattractant. Another more recent retrospective trial showed a correlation between hypocomplementemia and reduced progression-free and overall survival (145). These conflicting reports on the relationship between complement activation and PCNSL progression suggest a more nuanced role for complement in the natural history of disease, perhaps unique to different B cell lymphoma subtypes.

### Complement in Leptomeningeal Disease

Leptomeningeal disease (LMD), also known as leptomeningeal metastasis, describes the spread of metastatic neoplastic disease into the arachnoid mater, pia mater, and CSF (146). Approximately 5 to 8 percent of all cancer patients will develop LMD (147), with a uniformly dismal prognosis. Common cancers to present with LMD include acute lymphoblastic leukemia (ALL), breast cancer, lung cancer, melanoma, and non-Hodgkin lymphoma (146). Seeding of the leptomeninges can occur either *via* preexisting metastases within the CNS or *via* the extension of a tumor mass that abuts the meninges (148). The exact molecular pathophysiology of LMD dissemination is poorly understood, but it is thought to abide by the general principles of metastatic invasion, which include the potential for immune invasion and vascular remodeling (149).

Work from Boire et al. has demonstrated that complement protein C3 is upregulated in metastatic tumor cells and facilitates leptomeningeal spread. In xenograft and syngeneic murine models of breast and lung cancer, tissue samples from LMD demonstrated significantly increased C3 expression compared to samples from the primary tumors (65). Knockdown of C3 expression with intrathecal short hairpin RNA (shRNA) dramatically reduced metastasis to the leptomeninges in these murine models (see **Table 2**). In naïve mice, administration of exogenous C3a led to increased extravasation of intravenously administered dextran to the CSF, which implies that tumor-produced C3a might disrupt the blood-CSF barrier in order to facilitate leptomeningeal spread. Moreover, mice deficient in the receptor for the C3a anaphylatoxin showed decreased growth of cancer cells inoculated directly into the leptomeningeal space, suggesting that C3a also primes the CSF for tumor invasion. Analysis of CSF samples from patients with LMD also demonstrated increased C3 expression, and decreases in C2, C3, and C4 within the CSF correlated with response to intrathecal treatment in these patients.

#### Leptomeningeal Melanoma

The role of complement in leptomeningeal melanoma (LMM) is also an area of active research, but recent data suggest that complement activation is implicated in the natural history of the disease. Pathway enrichment on CSF samples collected from patients with LMM demonstrated upregulation in various proteins involved in the complement cascade for patients who were poor responders to treatment (66). These proteins include C2, C3, and those that form the MAC. Studies in syngeneic murine models of melanoma have shown that the C3a receptor is necessary for the growth and spread of disease (150). Specifically, they demonstrated that interaction with this receptor disrupts neutrophil and CD4+ T cell responses within the tumor microenvironment. More research is necessary to further characterize the biomolecular changes that underly the development of LMM and how complement is modulated throughout the course of disease.

### Complement in Radiation Necrosis

Radiation therapy (RT) remains a major component of the standard of care treatment for brain tumors. Despite significant improvement with the use of targeted and stereotactic radiosurgery to limit normal tissue toxicity, radiation necrosis (RN) remains a major adverse effect of RT. The incidence of RN has continued to increase given the more frequent use of adjuvant immunological therapy. RN is an undesirable, late exaggerated immune response to radiation-induced damage that results in progressive cerebral edema and peritumoral inflammation. Injury due to radiation can be further classified based on its spatial pattern (diffuse vs. focal) and the timing of injury in relation to treatment (acute, early delayed, and late) (151). Late radiation injury develops months to years after RT and is typically an irreversible and progressive phenomenon. Radiation exposure leads to endothelial cell injury, which manifests as dilated and thickened vessel walls. Along with astrocyte hyperplasia and hypertrophy, these changes are thought to comprise a “tissue injury unit” that precipitates eventual white matter necrosis (152, 153). Competing theories implicate oligodendrocyte damage and demyelination (154), autoimmune vasculitis (151), as well as changes in the fibrinolytic pathway (155) in the development of RN.

The role of complement in radiation-induced damage remains underexplored. Genetic studies in syngeneic mouse models of cancer and *ex vivo* human tumor samples have demonstrated that the C3a and C5a anaphylatoxins are upregulated in response to RT (156). In a murine model of melanoma, pretreatment of dexamethasone prior to RT reduced complement activation and reduced the efficacy of RT, as assessed by tumor volume measurements in the days post-RT (156). RT is known to engage the adaptive immune response (157, 158), so it is logical that elements of the complement cascade that bridge innate and adaptive immunity be engaged in this process.

As part of the stress response, RT also induces the exposure of damage-associated molecular patterns (DAMPs) and stress-related neo-epitopes following radiation-induced cell death. Exposure of these signals leads to a robust activation of the innate and adaptive immune system in the brain (101, 103, 159). Post-radiation inflammation likely involves locally activated and hematogenous-derived immune cells and components, given the effect of neoplastic pathology and radiation on blood brain barrier (BBB) integrity. The actual pathological pathways linking RT-induced cell stress, toxic edema, and cell death are still unknown. Consequently, there is a lack of targeted therapeutics to inhibit the initiation of this toxic neuroinflammation.

Prior work has demonstrated that exposure of DAMPs induces local activation of complement and deposition of C opsonins (i.e., C3b/C3d) in various disease models (160, 161). Relevant to malignancy and radiation exposure, prior work using a lymphoma model demonstrated that complement opsonins deposit locally after radiation injury and can be targeted by complement inhibitors to prevent the exacerbation of peritumoral inflammation (160). Studies in both rodents and nonhuman primates have demonstrated that complement proteins, including both opsonins and anaphylatoxins, as well as complement receptors are overexpressed and activated in the brain following radiation exposure (68, 69). Following its activation by radiation-induced stress, the complement system is then capable of self-amplification and robust activation of components of innate and adaptive immunity. This amplified activation is likely to promote worsening edema, mass effect, and neurodegeneration in peritumoral brain tissue.

## Therapeutic Modulation of the Complement System

Complement has been implicated in nearly all pathologies and disease of the CNS including vascular/ischemic stroke (1), autoimmune (162), and traumatic CNS pathologies (100). Despite the diverse pipeline of complement therapeutics developed over the past decade (159, 163), there are still limited complement inhibitors available to treat complement-related pathologies. To date, eculizumab, an anti-C5 antibody, along with its long-acting modification (i.e., Ultomiris) are the major success stories for complement based therapeutics. Initially approved to treat paroxysmal nocturnal hemoglobinuria and more recently approved for myasthenia gravis and AQp4-IgG positive neuromyelitis optica, anti-C5 acts systemically to inhibit complement activation at the level of the C5-convretase (163).

Although access to the BBB remains a major challenge in developing complement therapeutics, novel complement inhibitors that target different aspects of the cascade, C3 and C5 activation, are currently part of the treatment pipeline for CNS pathologies with age-related macular degeneration being the top targeted pathology. Although lampalizumab that targets the alternative pathway failed to show clinical benefit in macular degeneration, alternative inhibitors are still in different phases of clinical development and include: Compstatin (C3 inhibitor) along with its derivatives APL-1 and APL-2 (Apellis Pharmaceuticals), Mirococept (APT070, C3 inhibitor), PMX-53 (C5a receptor antagonist), C1-INH (inhibits classical and lectin pathways), Tesidolumab (inhibitor of C5 activation) among others (163).

In addition to the systemic therapeutics listed above, new generation of complement therapeutics are currently early in the translational spectrum and include tissue-targeted therapeutics that self-target to sites of active complement breakdown. In the setting of ischemic or traumatic brain injury, robust deposition of the complement breakdown product C3b/C3d provide a promising target to deliver therapeutics. As reviewed in (159), different generations of these targeted therapeutics have been described. Examples are complement receptor 2 (CR2) based inhibitors that constitute of fusion proteins of CR2 and inhibitors of one of different complement activation pathways; namely, CR2-fH, CR2-Crry and CR2-CD59 have been well studied in acute neurological pathologies such as stroke and traumatic brain injury. A similar approach include the tissue-targeted terminal pathway inhibitor CD59-2a-CRIg that uses the CRIg superfamily domains to target sites of C3 deposition and deliver complement inhibitor CD59 to the site of pathology. Finally, a different generation of targeted inhibitors applicable to CNS disease was designed using fusion proteins of single-chain variable fragments (scFv) of natural IgM antibodies that target stress-induced neo-epitopes fused to a complement inhibitor to allow its deliver to sites of active disease (159). This latter approach will have the added therapeutic value of inhibiting the antibody-based trigger of complement activation in addition to the targeted complement pathway.

Regarding complement modulation in the setting of CNS neoplastic disease, major frontiers in complement-targeted therapeutics in glioma are likely to include a focus on C1q-targeted therapeutics, given its consistent role in driving tumor progression, as well as C3-targeted therapeutics in the context of radiation to limit radiation-induced edema and degeneration allowing for maximal treatment dosing. Similarly, C3-targeted therapeutics (both C3 convertase inhibitors and C3a antagonists) are specifically attractive for metastatic disease given the role of C3 in driving edema after radiation, which is routinely used for these tumors, as well as the role of C3 in leptomeningeal spread. Systemic suppression of these complement components by therapeutics remains a major limitation due to limited bioavailability in the CNS and the risk of suppression of systemic defense mechanisms in a population known to be prone to infections. The use of tissue-targeted complement therapeutics [e.g., CR2-targeted or antibody targeted (159)] is one major avenue for overcoming these limitations.

## Conclusions

As presented in this review, complement modulation has been implicated in the development and progression of brain and spine tumors as well as cell injury in the peritumoral environment. As CNS neoplasms are a heterogeneous group of diseases, it follows that the implicated complement mechanisms are similarly diverse. The current body of evidence with regards to glioma supports predominantly inhibitory changes, in contrast to more recent evidence in non-CNS tumors that characterize complement activation as tumorigenic, whereas data in brain metastases, LMD, and RN present a mixed picture with regards to complement activation and inhibition. The proposed pathways in this review suggest novel diagnostic and therapeutic targets for a population of patients notoriously difficult to treat, both at initial diagnosis and with recurrence. This work also supports the potential role for targeted complement modulation in CNS tumors and emphasizes the need for more translational and preclinical studies in this field.

## Author Contributions

SY, AA, and KH conceptualized the scope of the review. SY performed the literature review. SY and AA wrote the initial manuscript. AA, ST, and KH reviewed and edited. All authors contributed to the article and approved the submitted version.

## Funding

SY is supported through grants from the National Institutes of Health (F30 CA216939, T32 GM008169). AA is supported through a grant from the Department of Veterans Affairs (I01 RX001141). KH is supported by a grant from the American Cancer Society.

## Conflict of Interest

The authors declare that the research was conducted in the absence of any commercial or financial relationships that could be construed as a potential conflict of interest.

## Publisher’s Note

All claims expressed in this article are solely those of the authors and do not necessarily represent those of their affiliated organizations, or those of the publisher, the editors and the reviewers. Any product that may be evaluated in this article, or claim that may be made by its manufacturer, is not guaranteed or endorsed by the publisher.
